# Effectiveness of Platelet-Rich Plasma Combined with Split-Thickness Skin Grafts for Skin Defects: A Systematic Review and Meta-Analysis

**DOI:** 10.3390/jcm15156003

**Published:** 2026-08-02

**Authors:** Rafael Dib Possiedi, Marcelo Augusto Fontenelle Ribeiro Junior, Lucas Fontenelle Vieira, Husna Irfan Thalib, Agata Grochowska-Krystman, Sariya Khan, Troy Shafer, Ayesha Jamal, Geovana Schulz, Jaques Waisberg

**Affiliations:** 1 Department of Surgery, R Adams Cowley Shock Trauma Center, University of Maryland, Baltimore, MD 21201, USA; rpossiedi@som.umaryland.edu (R.D.P.); troydshafer@gmail.com (T.S.); 2 Programa de Pós-Graduação Stricto Sensu em Ciências da Saúde, Department of Surgery, Hospital do Servidor Público Estadual, Instituto de Assistência Médica ao Servidor Público Estadual (IAMSPE), São Paulo 04029-000, SP, Brazil; jaqueswaisberg@uol.com.br; 3 Department of Surgery, Médecins Sans Frontières, 1050 Brussels, Belgium; 4 Department of Surgery, Pontifical Catholic University of São Paulo, Campus Sorocaba, Sorocaba 18030-070, SP, Brazil; 5 Department of Surgery, Florida Atlantic University Charles E Schmidt College of Medicine, Boca Raton, FL 33431, USA; lucaovieira10@gmail.com; 6 General Medicine Practice Program, School of Medicine, Batterjee Medical College, Jeddah 21442, Saudi Arabia; husnairfan2905@gmail.com (H.I.T.); sariyak2003@gmail.com (S.K.); drayeshajamal1@gmail.com (A.J.); 7 Faculty of Medicine, Medical University of Lodz, 90-153 Lodz, Łódź Voivodeship, Poland; agatagrch@gmail.com; 8 School of Medicine, Pontifical Catholic University of Paraná, Curitiba 80215-901, PR, Brazil; geovanaschulz.med@gmail.com; 9 Department of Surgery, Centro Universitário Faculdade de Medicina do ABC, Santo André 09060-870, SP, Brazil

**Keywords:** platelet-rich plasma, split-thickness skin graft, wound healing, skin graft take, hematoma, graft loss, meta-analysis, systematic review

## Abstract

**Background/Objectives:** Platelet-rich plasma (PRP) has been proposed as an adjunct to split-thickness skin grafting (STSG) to enhance graft adherence and reduce complications. However, evidence remains heterogeneous. This study evaluated the effectiveness of PRP combined with STSG compared with STSG alone. **Methods:** A systematic review and meta-analysis was conducted following PRISMA guidelines. Databases and trial registries were searched for randomized controlled trials and comparative observational studies. Ten studies were included. The primary outcome was skin graft take. Secondary outcomes were wound healing time, graft loss, and hematoma incidence. Random-effects models were used. Risk of bias was assessed with RoB 2 and ROBINS-I, and certainty of evidence with GRADE. **Results:** Ten studies (7 randomized controlled trials and 3 observational studies) were included. PRP improved graft take (mean difference 9.93%; 95% CI: 4.70–15.17; *p* = 0.0002; I^2^ = 75%), with a larger effect in patient-level analyses (14.78; 95% CI: 10.97–18.59; *p* < 0.00001; I^2^ = 0%). Healing time could not be pooled. Hematoma incidence was reduced (risk ratio 0.26; 95% CI: 0.14–0.48; *p* < 0.0001; I^2^ = 0%). Graft loss was reduced in parallel-group trials (risk ratio 0.26; 95% CI: 0.11–0.58; *p* = 0.001; I^2^ = 0%) but not in sensitivity analysis (risk ratio 0.35; 95% CI: 0.10–1.19; *p* = 0.09; I^2^ = 73%). Evidence certainty was low to very low. **Conclusions:** PRP may improve graft take and reduce hematoma after STSG, but effects on graft loss and healing time remain uncertain.

## 1. Introduction

Skin grafting remains a widely used technique for the management of soft tissue defects, particularly in conditions such as burns, chronic ulcers, and traumatic wounds. Split-thickness skin grafts (STSGs) are commonly applied due to their relative simplicity and effectiveness in resurfacing large defects. The process of graft healing involves sequential phases including adhesion, inosculation, and neoangiogenesis, which are critical for successful graft integration [1].

The success of STSG is influenced by several local factors at the recipient site. Mechanical instability, fluid accumulation, and infection can impair graft adherence and compromise graft take. Conventional fixation methods such as sutures and staples aim to reduce graft mobility and prevent seroma formation, but may not fully address early biological processes involved in graft integration [1].

Platelet-rich plasma (PRP) has been proposed as an adjunctive therapy in skin grafting due to its biological properties. PRP is an autologous concentration of platelets containing growth factors such as platelet-derived growth factor, transforming growth factor-β, vascular endothelial growth factor, and epidermal growth factor, which are involved in tissue repair and regeneration [1,2].

In addition to its biological effects, PRP may contribute to graft adherence through its fibrin content and may be associated with enhanced revascularization via growth factor release. These mechanisms provide a theoretical basis for improving graft fixation and subsequent graft take when PRP is applied to the recipient site prior to graft placement [2].

Recent clinical studies have reported improved outcomes with the use of PRP in STSG. A prospective comparative study demonstrated significantly higher graft uptake at early postoperative time points and reduced graft edema and hematoma formation in the PRP group compared to conventional methods [2]. Similarly, other prospective studies have shown improved graft adherence, reduced seroma and hematoma formation, and decreased donor-site morbidity, including reduced pain and improved healing [3].

However, findings across studies remain heterogeneous. Included populations vary widely in terms of wound etiology, including burns, trauma, and chronic ulcers, and there is substantial variability in PRP preparation techniques, application methods, and timing of outcome assessment [2,3]. In addition, some studies are designed at the wound level rather than the patient level, allowing the same patient to contribute to both intervention and control groups, which introduces potential unit-of-analysis bias [1].

Previous meta-analyses have attempted to synthesize this evidence; however, important challenges remain in the interpretation of pooled estimates. Heterogeneous clinical contexts, variable PRP preparation protocols, and inconsistent outcome definitions have been combined across studies, which may limit the ability to fully account for this variability. Studies with fundamentally different designs, including intra-patient and parallel-group trials, have been analyzed within the same framework, which may influence pooled estimates when unit-of-analysis differences are not consistently addressed. In addition, interventions with distinct mechanisms, such as topical PRP application and intralesional PRP injection [4], have also been included within the same analytical frameworks, despite potential differences in clinical applicability [5].

Similarly, available meta-analytic evidence is based on a limited number of studies with heterogeneous populations and outcome reporting. Analyses relying on small datasets and variability in study design and intervention characteristics may limit the robustness and interpretability of pooled estimates [6].

Moreover, recent evidence highlights persistent gaps in high-quality data, particularly in burn populations. Ongoing randomized trials emphasize the need for adequately powered, multicenter studies with standardized outcome definitions, including graft take and clinically relevant endpoints, to better establish the role of PRP in STSG [7].

Given these limitations, a more cautious and clinically coherent synthesis is warranted. The present systematic review and meta-analysis aims to evaluate the effectiveness of PRP as an adjunct to STSG with a focus on clinically relevant and comparable outcomes and transparent consideration of study design differences.

## 2. Materials and Methods

This systematic review and meta-analysis was conducted and reported in accordance with the Cochrane Handbook for Systematic Reviews of Interventions and the Preferred Reporting Items for Systematic Reviews and Meta-Analyses (PRISMA) guidelines [8,9]. Studies were selected according to the population, intervention, comparator, outcomes, timing, and study design (PICOTT) framework.

The population consisted of patients with skin defects requiring reconstruction with split-thickness skin grafts (STSGs), including wounds resulting from burns, trauma, necrotizing soft tissue infections, or other acute tissue loss requiring grafting. The intervention of interest was the application of platelet-rich plasma (PRP), either autologous or allogeneic, with or without thrombin gel, in conjunction with STSG.

The comparator consisted of conventional STSG without PRP application, performed using standard fixation methods and dressings.

The primary outcome was skin graft take or graft success rate, as reported by the included studies. Secondary outcomes included complete wound healing time, defined as the time to full epithelialization when reported, skin graft loss, and postoperative complications such as hematoma formation, infection, or other graft-related adverse events.

Eligible study designs included randomized controlled trials and observational comparative studies, including prospective or retrospective cohort studies. Studies were eligible when they reported comparative outcomes between PRP-augmented STSG and conventional STSG with extractable numerical data. No restrictions were placed on publication date or duration of follow-up.

Studies were excluded if they did not involve STSG procedures, did not include a comparator group treated with conventional STSG alone, or did not report outcomes relevant to the objectives of this review. Studies using PRP via intralesional injection were excluded due to a different mechanism of action compared with topical application at the graft bed. Additional exclusion criteria included case reports, case series with fewer than five patients, review articles, editorials, animal studies, and conference abstracts without extractable data. Studies in which outcomes could not be separated between treatment groups were also excluded. No language restrictions were applied when sufficient data could be extracted from the full text or English abstract.

The methodological approach and eligibility criteria followed a predefined protocol developed prior to study initiation.

### 2.1. Protocol Registration

This systematic review and meta-analysis was performed in accordance with a previously established protocol registered in the International Prospective Register of Systematic Reviews (PROSPERO) [10] on March 2026 (registration number CRD420261353493), to ensure methodological transparency and rigor.

### 2.2. Search Strategy

A comprehensive literature search was independently conducted by two reviewers across several electronic databases, including PubMed/MEDLINE, Embase, ICTRP, Web of Science, and the Cochrane Central Register of Controlled Trials, Clinical trials.gov. The search strategy combined controlled vocabulary and free-text terms related to platelet-rich plasma and skin grafting. Keywords included variations of “platelet-rich plasma”, “PRP”, “autologous platelet-rich plasma”, “split-thickness skin graft”, “STSG”, and “skin grafting”. The search strategy was adapted for each database according to its indexing system, and the complete search strings are provided in the Supplementary Materials.

To ensure comprehensive coverage, the reference lists of all included articles and relevant reviews were manually screened to identify additional potentially eligible studies. Study eligibility was determined according to the predefined inclusion and exclusion criteria described in the study protocol.

Two reviewers independently evaluated titles and abstracts during the initial screening phase, followed by full-text assessment of potentially relevant studies. Inter-reviewer agreement during study selection was evaluated using the Cohen’s kappa coefficient (κ). Any disagreements were resolved through discussion, and when necessary, a senior investigator made the final decision regarding study inclusion.

### 2.3. Data Extraction

Two reviewers independently screened titles, abstracts, and full-text articles using the Rayyan^®^ [11] platform according to the predefined eligibility criteria established in the study protocol. Any discrepancies during the selection process were resolved through discussion, and when consensus could not be reached, a third reviewer was consulted.

Data extraction was performed independently by two investigators using a standardized data collection form developed specifically for this review. The following study characteristics were recorded: first author, year of publication, country of origin, and institution or department where the study was conducted to identify potential overlap of patient populations. Additional methodological details included study design and the total number of participants allocated to the PRP + STSG group and the conventional STSG group.

Baseline patient characteristics were collected when available, including age, sex distribution, relevant comorbidities, etiology of the wound (e.g., burns, trauma, or necrotizing soft tissue infections), and other reported clinical variables related to wound severity.

Details regarding treatment strategies were also extracted. For the intervention group, information related to platelet-rich plasma preparation and application methods was collected when reported. For the comparator group, details regarding conventional split-thickness skin graft fixation techniques and standard wound management approaches were recorded.

The outcomes of interest included complete wound healing time, skin graft take rate, skin graft loss, and postoperative complications such as hematoma or infection. Follow-up duration and postoperative observations were also documented when reported.

When outcome data were reported narratively or embedded within tables, numerical values were extracted whenever they could be clearly distinguished between the intervention and comparator groups. When necessary, standard deviations were derived from reported standard errors.

Only outcomes that were reported with sufficiently comparable definitions and adequate numerical detail in at least two studies were considered eligible for quantitative synthesis. Outcomes with substantial variability in definitions or insufficient numerical information were not pooled and were instead described narratively.

### 2.4. Risk of Bias Assessment

The methodological quality of the included studies was evaluated independently by two reviewers using validated tools appropriate to each study design. Randomized controlled trials were assessed using the Cochrane Risk of Bias 2 (RoB 2) tool, while non-randomized studies were evaluated using the Risk of Bias In Non-randomized Studies of Interventions (ROBINS-I) framework.

For randomized trials, the RoB 2 tool was applied across the following domains: bias arising from the randomization process, bias due to deviations from intended interventions, bias related to missing outcome data, bias in the measurement of outcomes, and bias in the selection of the reported results. Each domain was judged as low risk of bias, some concerns, or high risk of bias, according to Cochrane guidance.

Observational studies were evaluated using the ROBINS-I instrument, which examines seven methodological domains: bias due to confounding, selection of participants, classification of interventions, deviations from intended interventions, missing data, measurement of outcomes, and selection of reported results. Particular attention was given to potential confounding factors related to wound etiology, baseline wound severity, and patient comorbidities, which could influence graft outcomes independently of the intervention.

Each domain was graded as low, moderate, serious, or critical risk of bias, and an overall risk of bias judgment was assigned in accordance with ROBINS-I recommendations.

To ensure consistency in the evaluation process, reviewers initially calibrated their assessments using a subset of studies. Disagreements during the evaluation process were resolved through discussion, and when necessary, a senior investigator was consulted to reach a final decision.

### 2.5. Statistical Analysis

Statistical analyses were performed using random-effects models to account for expected clinical and methodological heterogeneity among studies. All analyses and forest plots were generated using Review Manager (RevMan) software, Version 10.0.0 [12].

For dichotomous outcomes, pooled effect estimates were calculated as risk ratios (RR) with 95% confidence intervals (CI) using the Mantel–Haenszel method. Continuous outcomes were analyzed as mean differences (MD) with 95% confidence intervals using the inverse variance method. Statistical significance was defined when confidence intervals did not cross the null value.

Between-study heterogeneity was assessed using the I^2^ statistic and the between-study variance (τ^2^). The I^2^ statistic represents the proportion of total variability attributable to heterogeneity rather than chance.

Predefined subgroup analyses were planned according to study design. Subgroup analysis was performed for hematoma incidence, stratifying studies into randomized controlled trials and observational studies, as sufficient data were available. Subgroup analyses were not feasible for other outcomes due to limited data.

Sensitivity analyses were performed to evaluate the robustness of the pooled estimates. For skin graft take, analyses were repeated including only studies with independent patient-level allocation. For graft loss, the primary analysis was restricted to parallel-group randomized controlled trials, and an additional sensitivity analysis incorporated split-wound trials together with parallel-group studies.

Because some included trials used intra-patient or split-wound designs, which may introduce within-patient correlation, these studies were included as reported when the information required to adjust for clustering was unavailable. Sensitivity analyses were therefore used to assess the potential influence of these designs on pooled estimates.

### 2.6. Assessment of Certainty of Evidence (GRADE)

The certainty of the evidence was evaluated using the GRADE approach. Four domains were considered in this assessment: risk of bias, inconsistency, indirectness, and imprecision. Publication bias was not formally assessed due to the limited number of studies available for each outcome. For non-randomized observational studies, including retrospective cohort studies, the initial level of certainty was assigned according to GRADE recommendations for this study design, with further downgrading applied when concerns were identified in any of the evaluated domains. The overall certainty of evidence was determined separately for each outcome.

## 3. Results

### 3.1. Search Process

The database and registry search identified a total of 466 records. After removal of 43 duplicate records, 423 titles and abstracts were screened, of which 384 were excluded during title and abstract screening. Thirty-nine reports were retrieved for full-text assessment. Following full-text evaluation, 29 studies were excluded because of ineligible study design (*n* = 24) or insufficient outcome data for quantitative extraction (*n* = 5).

A total of 10 studies were included in the systematic review and meta-analysis [1,2,3,13,14,15,16,17,18,19]. Among them, 7 were randomized controlled trials [1,14,15,16,17,18,19], whereas 3 were observational comparative studies [2,3,13]. Inter-reviewer agreement was assessed using Cohen’s kappa. Cohen’s kappa was 0.36 for title and abstract screening (98.5% observed agreement) and 0.95 for full-text screening (97.4% observed agreement).

**Figure 1:**
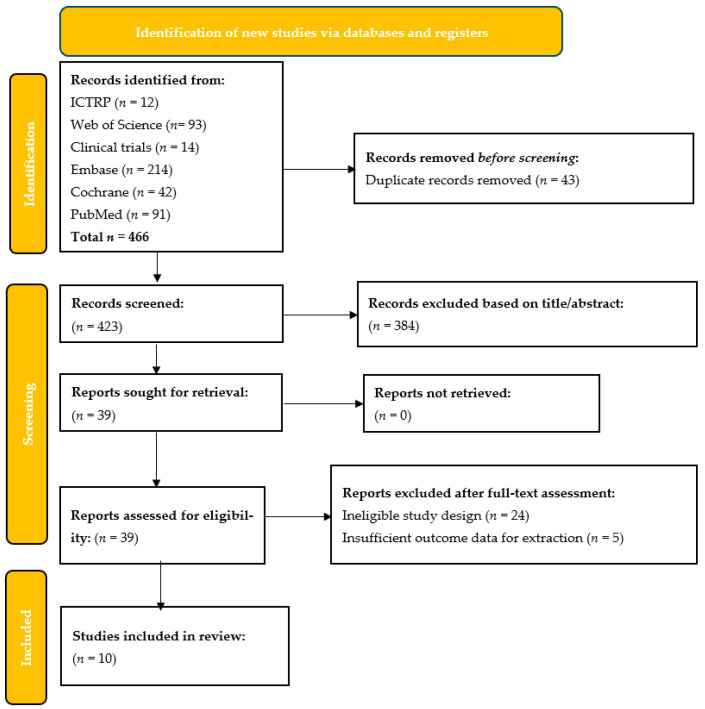
PRISMA 2020 flow diagram illustrating the study selection process for the systematic review and meta-analysis of platelet-rich plasma-augmented split-thickness skin grafting versus conventional grafting.

The characteristics of the included studies are summarized in Table S1, and the study selection process is presented in Figure 1.

### 3.2. Skin Graft Take

**Figure 2:**
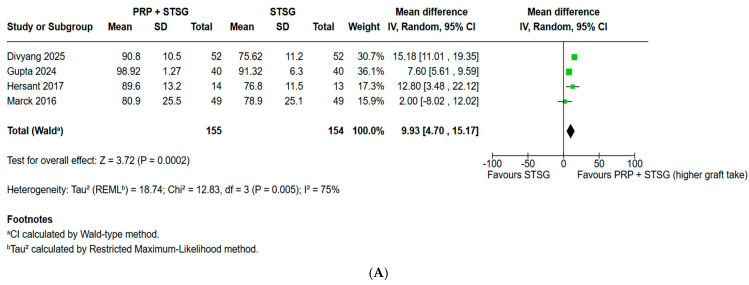

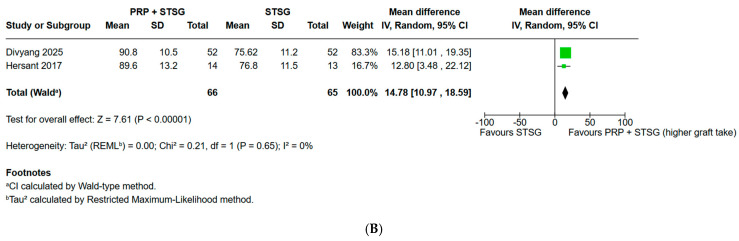
Forest plots of skin graft take comparing platelet-rich plasma–augmented split-thickness skin grafting versus conventional split-thickness skin grafting. (**A**) Primary analysis including all eligible studies. (**B**) Sensitivity analysis restricted to patient-level studies.

Four studies were included in the quantitative synthesis evaluating skin graft take after split-thickness skin grafting augmented with PRP. The pooled analysis demonstrated a significantly higher graft take in the PRP + STSG group compared with conventional STSG alone (mean difference [MD] 9.93; 95% CI 4.70–15.17; *p* = 0.0002) (Figure 2A), with heterogeneity across studies (I^2^ = 75%) [1,2,15,16].

A sensitivity analysis restricted to studies with patient-level allocation included two studies and showed a significant improvement in graft take with PRP augmentation (MD 14.78; 95% CI 10.97–18.59; *p* < 0.00001), with no observed heterogeneity (I^2^ = 0%) (Figure 2B) [2,16]. This reduction in heterogeneity suggests that intra-patient designs may have contributed to heterogeneity due to lack of adjustment for within-patient correlation.

### 3.3. Complete Wound Healing Time

This outcome was reported in a very limited number of studies. Only one randomized controlled trial provided extractable and directly comparable data, defining complete healing as 100% re-epithelialization and reporting time to full wound closure [16]. One additional study reported time to complete healing using an intra-patient design with limited methodological standardization, precluding quantitative pooling [13]. The remaining studies did not assess this outcome. Due to the limited number of studies, differences in study design and reporting, and insufficient comparable data, this outcome did not meet predefined criteria for quantitative synthesis, restricting the ability to draw conclusions.

### 3.4. Hematoma

Six studies were included in the meta-analysis evaluating hematoma incidence following split-thickness skin grafting with adjunctive PRP. Overall, PRP use was associated with a significantly lower risk of hematoma compared with conventional STSG (risk ratio [RR] 0.26; 95% CI 0.14–0.48; *p* < 0.0001), with no observed heterogeneity across studies (I^2^ = 0%) [1,2,3,14,17,18].

**Figure 3:**
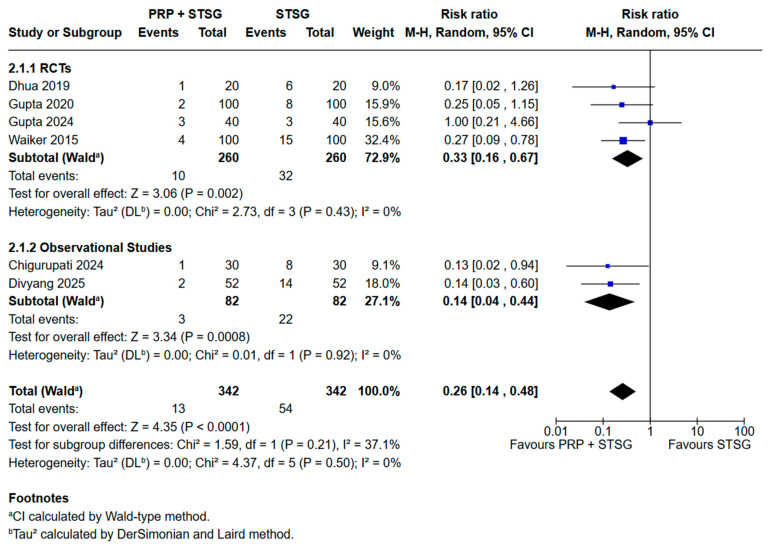
Forest plot of hematoma incidence following split-thickness skin grafting with adjunctive platelet-rich plasma versus conventional grafting, with subgroup analysis according to study design.

Subgroup analysis according to study design showed consistent results. Among randomized controlled trials, PRP significantly reduced hematoma incidence (RR 0.33; 95% CI 0.16–0.67; *p* = 0.002; I^2^ = 0%) [1,14,17,18]. Similarly, observational studies demonstrated a significant reduction in hematoma risk with PRP augmentation (RR 0.14; 95% CI 0.04–0.44; *p* = 0.0008; I^2^ = 0%) (Figure 3) [2,3].

No significant subgroup differences were observed between randomized and observational studies (*p* = 0.21), indicating consistent treatment effects across study designs.

### 3.5. Skin Graft Loss

**Figure 4:**
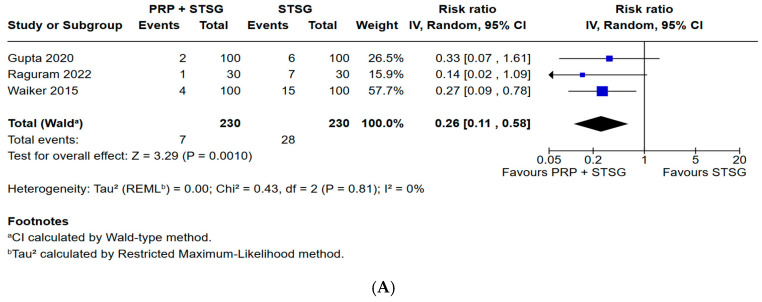

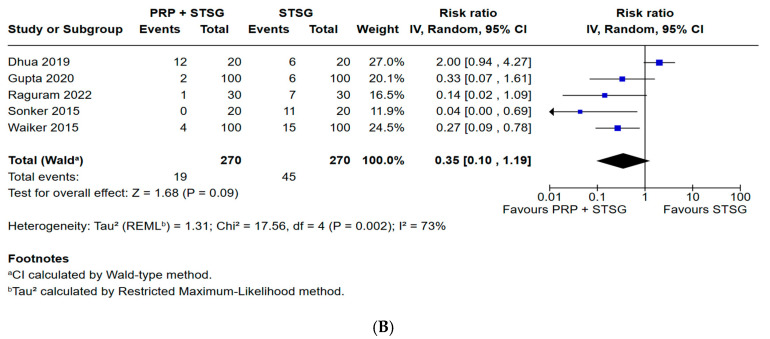
Forest plots of graft loss incidence comparing platelet-rich plasma–augmented split-thickness skin grafting versus conventional grafting. (**A**) Primary analysis including parallel-group randomized controlled trials. (**B**) Sensitivity analysis including both parallel-group and split-wound randomized trials.

Three parallel-group randomized controlled trials were included in the primary meta-analysis evaluating graft loss following split-thickness skin grafting with adjunctive platelet-rich plasma (PRP). The pooled analysis demonstrated a significantly lower risk of graft loss in the PRP + STSG group compared with conventional STSG (risk ratio [RR] 0.26; 95% CI 0.11–0.58; *p* = 0.001), with no observed heterogeneity among studies (I^2^ = 0%) (Figure 4A) [14,18,19].

A sensitivity analysis including both parallel-group and split-wound randomized trials was subsequently performed, incorporating five studies. In this analysis, the pooled estimate showed no statistically significant difference between groups (RR 0.35; 95% CI 0.10–1.19; *p* = 0.09), with substantial heterogeneity observed across studies (I^2^ = 73%) (Figure 4B) [13,14,17,18,19]. The loss of statistical significance and the increase in heterogeneity following the inclusion of split-wound trials suggest that the inclusion of intra-patient (split-wound) designs may have influenced the pooled estimates due to lack of adjustment for within-patient correlation. These findings indicate sensitivity of the results to study design.

### 3.6. Risk of Bias

Seven randomized controlled trials were assessed using the RoB 2 tool [1,14,15,16,17,18,19]. The overall risk of bias was judged as “some concerns” in all included trials. Bias arising from the randomization process was rated as low in two studies [15,16] and as some concerns in the remaining trials [1,14,17,18,19].

Bias due to deviations from intended interventions was rated as low in one study [15] and as some concerns in the others [1,14,16,17,18,19]. Bias due to missing outcome data was low in six studies [1,14,16,17,18,19] and was judged as some concerns in one study [15]. Bias in measurement of the outcome was rated as low in three studies [15,16,19] and as some concerns in the remaining trials [1,14,17,18]. Bias in selection of the reported results was rated as low in one study [15] and as some concerns in the others [1,14,16,17,18,19]. A detailed domain-level assessment is presented in Table S2.

Three observational studies were assessed using the ROBINS-I tool [2,3,13]. Two studies were judged to have a serious overall risk of bias [3,13], and one study was classified as having a moderate overall risk of bias [2].

Across studies, bias due to confounding was rated as serious in two studies [3,13] and moderate in one [2]. Bias in selection of participants was rated as moderate in two studies [3,13] and low in one [2]. Bias in classification of interventions was low in all studies [2,3,13]. Bias due to deviations from intended interventions was rated as moderate across all studies [2,3,13].

Bias due to missing data was low in all studies [2,3,13]. Bias in measurement of outcomes was rated as moderate in all studies [2,3,13]. Bias in selection of the reported result was judged as moderate across all studies [2,3,13]. A detailed domain-level assessment is presented in Table S3.

### 3.7. Certainty of Evidence (GRADE) Across Outcomes

Publication bias was not formally assessed for any outcome due to the limited number of included studies.

The overall certainty of evidence for skin graft take was judged to be very low. This rating was downgraded due to serious risk of bias arising from methodological limitations across randomized controlled trials [1,15,16] and the inclusion of an observational study [2] with moderate risk of bias. Additional downgrading was applied for serious inconsistency, reflecting substantial heterogeneity in the primary analysis, and for serious imprecision due to the limited number of studies and relatively small sample sizes. No concerns regarding indirectness were identified, as the evidence was directly applicable to the research question.

For hematoma, the certainty of evidence was considered low. Downgrading was applied for serious risk of bias, reflecting methodological limitations across both randomized [1,14,17,18] and observational studies [2,3], and for serious imprecision related to the limited number of studies and overall sample size. No serious concerns were identified with respect to inconsistency or indirectness.

With regard to skin graft loss, the certainty of evidence was also rated as low. This was downgraded for serious risk of bias due to limitations in randomized controlled trials [14,17,18,19] and the inclusion of an observational study [13] with serious risk of bias. A further downgrade for serious imprecision was applied due to the limited number of studies and relatively small sample sizes. No serious concerns regarding inconsistency or indirectness were identified, as findings were consistent across studies and directly applicable to the research question. A detailed *certainty of evidence (GRADE) across outcomes* is presented in Table S4.

## 4. Discussion

This systematic review and meta-analysis evaluated the role of platelet-rich plasma (PRP) as an adjunct to split-thickness skin grafting (STSG). The findings suggest that PRP is associated with improvements in graft take and a reduction in hematoma formation. However, the effect on graft loss was not consistent across analyses, and evidence for complete wound healing time remains insufficient.

The observed improvement in graft take across studies [1,2,15,16] should be interpreted in the context of substantial heterogeneity and very low certainty of evidence. Importantly, when the analysis was restricted to studies with patient-level allocation, the number of contributing studies was markedly reduced [2,16]. These considerations indicate that, although a potential benefit may exist, the substantial heterogeneity, very low certainty of evidence, and limited number of studies preclude firm conclusions regarding the magnitude of effect.

In contrast, the reduction in hematoma incidence was consistently observed across all included studies evaluating this outcome [1,2,3,14,17,18], with low statistical heterogeneity. This consistency across studies strengthens confidence in the direction of the effect, although the certainty of evidence remains low. Among the evaluated outcomes, hematoma reduction represents the most stable finding in the current evidence base. The consistent reduction in hematoma formation observed across studies is biologically plausible given the central role of platelets in hemostasis. PRP delivers a supraphysiologic concentration of platelets to the wound bed, enhancing the availability of platelet-derived coagulation mediators. Upon vascular injury, activated platelets adhere to exposed subendothelial collagen through von Willebrand factor, aggregate to form the primary hemostatic plug, and interact with fibrin to stabilize clot formation. In addition, platelet α-granules contain fibrinogen, von Willebrand factor, and coagulation factors V, XI, XIII, and prothrombin, which contribute to clot formation and stabilization. Collectively, these mechanisms provide a biologically plausible explanation for the lower incidence of postoperative hematoma observed in the included studies, although this hypothesis has not been directly evaluated in skin grafting procedures [20].

The effect of PRP on graft loss differed according to study design. A reduction in graft loss was observed in analyses restricted to parallel-group randomized controlled trials [14,18,19], but this effect was not maintained when intra-patient (split-wound) studies were included [13,14,17,18,19]. This divergence highlights the influence of study design on effect estimates and underscores the importance of accounting for unit-of-analysis issues when interpreting pooled results. In this context, estimates derived from parallel-group trials may provide estimates less affected by unit-of-analysis bias, whereas analyses including intra-patient designs should be considered exploratory. The inability to adjust for within-patient correlation represents a limitation of the present analysis and may have affected the precision of pooled estimates.

An important observation from this review is the limited and inconsistent reporting of complete wound healing time. Only one study provided extractable comparative data for this outcome [16], while other studies reported healing time descriptively or used intra-patient designs that precluded quantitative pooling [13]. This limits the ability to assess a clinically relevant endpoint.

Compared with previous meta-analyses, the present study adopts a more conservative interpretation of the available evidence. Prior analyses have reported favorable effects of PRP on graft-related outcomes; however, these conclusions were often derived from pooled estimates that did not consistently account for key sources of methodological heterogeneity. In particular, differences in study design, including the combination of intra-patient and parallel-group trials within the same analytical framework, were not systematically addressed, which may influence the interpretation of pooled effect estimates [5,6].

In the present study, these methodological considerations were explicitly incorporated into the interpretation of the findings. Although the limited number of studies and variability in reporting precluded full statistical adjustment for these factors, the results were interpreted conservatively, acknowledging the potential influence of heterogeneity and study design on the observed effects rather than assuming a consistent treatment benefit.

These findings have important implications for both research and clinical practice. Rather than confirming a broad clinical benefit of PRP, this review indicates that hematoma reduction is the most consistent finding across the available studies, whereas improvements in graft take, graft loss, and wound healing remain uncertain because of methodological limitations and the low to very low certainty of the available evidence. This more balanced interpretation may help clinicians make informed decisions regarding the selective rather than routine use of PRP in split-thickness skin grafting.

Several limitations should be acknowledged. Methodological heterogeneity across studies, particularly regarding study design, may have influenced pooled estimates. Clinical heterogeneity was also present, as studies included patients with markedly different wound etiologies (e.g., burns, trauma, chronic ulcers, and necrotizing infections), which may influence treatment response and limit comparability across studies. In addition, variability in PRP preparation and application protocols was observed across studies, including differences in centrifugation methods (single vs. double spin), activation techniques (e.g., thrombin or calcium chloride), and formulation, which may have contributed to differences in observed outcomes. Additionally, one included study used allogeneic PRP [13], whereas the remaining nine studies used autologous PRP. This difference in PRP source may introduce additional heterogeneity in biological activity and treatment response and represents a deviation from a purely autologous intervention.

Finally, the overall certainty of evidence ranged from low to very low across outcomes. This indicates that the true effect may differ from the observed estimates and raises the possibility that the observed effects may be overestimated.

From a clinical perspective, the current evidence does not support the routine use of PRP as an adjunct to split-thickness skin grafting. However, the consistent reduction in hematoma incidence observed across studies suggests that PRP may be beneficial in selected clinical scenarios where minimizing postoperative hematoma is considered particularly important. Further high-quality randomized controlled trials with standardized methodologies and outcome reporting are required to clarify its role.

## 5. Conclusions

Platelet-rich plasma used as an adjunct to split-thickness skin grafting was associated with improved graft take and reduced hematoma incidence. However, these findings should be interpreted with caution due to substantial heterogeneity, limitations related to study design, and low to very low certainty of evidence. The effect on graft loss appears to be influenced by methodological differences across studies, and evidence for complete wound healing time remains insufficient. Current data do not support definitive conclusions regarding the magnitude of benefit or routine clinical use. Further high-quality, adequately powered randomized controlled trials with standardized methodologies and outcome reporting are required to clarify the role of platelet-rich plasma in split-thickness skin grafting.

## Data Availability

The results of this study are based exclusively on data extracted from previously published studies, all of which are properly cited in the manuscript. No original datasets were generated or analyzed. Therefore, data sharing is not applicable.

## Supplementary Materials

The following supporting information can be downloaded at https://www.mdpi.com/article/10.3390/jcm15156003/s1, Table S1: Baseline characteristics of included studies; Table S2: Risk of bias assessment of randomized controlled trials (RoB 2); Table S3: Risk-of-bias assessment of observational studies (ROBINS-I); Table S4: GRADE assessment of certainty of evidence for outcomes; and PRISMA 2020 Checklist.

## Abbreviations

The following abbreviations are used in this manuscript:

CI	Confidence Interval
CPD-A	Citrate Phosphate Dextrose Adenine
GRADE	Grading of Recommendations Assessment, Development, and Evaluation
I^2^	Inconsistency index
ICTRP	International Clinical Trials Registry Platform
MD	Mean Difference
PRISMA	Preferred Reporting Items for Systematic Reviews and Meta-Analyses
PROSPERO	International Prospective Register of Systematic Reviews
PRP	Platelet-Rich Plasma
RCT	Randomized controlled trial
ROBINS-I	Risk Of Bias In Non-randomized Studies of Interventions
RoB 2	Risk of Bias 2 (Revised Cochrane risk-of-bias tool for randomized trials)
RR	Risk ratio
SD	Standard deviation
STSG	Split-Thickness Skin Graft
RPM	revolutions per minute
Z	Z-score
