# Supplementary material for: Effectiveness of Platelet-Rich Plasma Combined with Split-Thickness Skin Grafts for Skin Defects: A Systematic Review and Meta-Analysis
Source: J Clin Med. 2026 Aug 2;15(15):6003. doi: 10.3390/jcm15156003 (PMC13467570; doi:10.3390/jcm15156003)
Supplement: Supplementary file 1 [file jcm-15-06003-s001.zip › jcm-4452451-supplementary.pdf]

SUPPLEMENTARY MATERIAL

Search Terms

PubMed Search Strategy: *(("autologous platelet-rich plasma" OR "platelet-rich plasma" OR "A-PRP" OR "PRP" OR "thrombin gel") AND ("split-thickness skin graft" OR "STSG" OR "skin graft" OR "skin grafting"))*

Cochrane: *(("autologous platelet-rich plasma" OR "platelet-rich plasma" OR "A-PRP" OR "PRP" OR "thrombin gel") AND ("split-thickness skin graft" OR "STSG" OR "skin graft" OR "skin grafting"))*

Embase: *('platelet rich plasma'/exp OR 'platelet rich plasma':ti,ab OR prp:ti,ab OR 'autologous platelet rich plasma':ti,ab OR 'platelet gel':ti,ab) AND ('skin graft'/exp OR 'split thickness skin graft':ti,ab OR stsg:ti,ab OR 'skin grafting':ti,ab OR ((skin NEAR/3 graft*):ti,ab)) AND (stsg:ti,ab OR split-thickness:ti,ab OR graft*:ti,ab) NOT (review:it OR editorial:it OR letter:it)*

Web of Science: *(("platelet rich plasma" OR "platelet-rich plasma" OR "autologous platelet rich plasma" OR "autologous platelet-rich plasma") AND ("split thickness skin graft" OR "split-thickness skin graft" OR STSG)) AND (graft OR grafting)*

ICTRP: *(("autologous platelet-rich plasma" OR "platelet-rich plasma" OR "A-PRP" OR "PRP" OR "thrombin gel") AND ("split-thickness skin graft" OR "STSG" OR "skin graft" OR "skin grafting"))*

ClinicalTrials.gov: *(platelet rich plasma OR PRP) AND ("split thickness skin graft" OR STSG OR "skin graft")*

Table S1- Baseline characteristics of included studies

Study/ Year	Type of Study	Comparison design	No. of patients (PRP / Control)	Age Years (±sd)†	Male, n (%)	Population	PRP preparation	PRP Application	STSG alone	Mesh ratio of STSG	Follow-up, days
**Chigurupati 2024**	Prospective interventional study (non-randomized controlled)	Parallel patient groups (patient-level comparison)	30/30	Not reported	39 (65)	Acute & chronic wounds (trauma, Burns, malignancy, benign)	Double centrifugation	Topical before STSG	STSG + sutures/staples	Not reported	3 months
**Dhua 2019**	RCT	Parallel patient groups (patient-level comparison)	20/20	35.5	24 (60)	Traumatic, infective, post-burn wounds	Double centrifugation PRP with $CaCl_2$ activation	Topical before STSG	STSG + sutures/staples	Not reported	3 months
**Divyang 2025**	Prospective comparative	Parallel patient groups (patient-level comparison)	52/52	41-80 range	66 (63.43)	necrotizing fasciitis, diabetic foot ulcers, burns, trauma	Double centrifugation	Topical before STSG	STSG alone	Not reported	7 days
**Gupta 2020**	RCT	Parallel patient groups (patient-level	100/100	PRP: 45.1 / control: 48.2	107 (53.5)	Burns & healing ulcers	Single centrifugation method	Thin PRP layer before graft	Standard STSG	Not reported	6 days

		comparison)									
**Hersant 2017**	RCT	Parallel patient groups (patient-level comparison)	14/13	PRP: 54.8±19. 8 vs control: 57.7±15. 8	Not reporte d	Necrotizing fasciitis / Fournier gangrene	RegenKit PRP with thrombin activation	Sprayed on wound bed and graft	STSG alone	1:1.5	Until healing (~37.9 vs 73.7 days)
**Raguram 2022**	RCT	Parallel patient groups (patient-level comparison)	30/30	Not reporte d	Not reporte d	Chronic ulcers, burns, trauma	Autologous PRP (CPD-A anticoagulant)	Topical before STSG	STSG + sutures	Not reporte d	~10–15 days
**Marck 2016**	RCT	Intra-patient (each patient contributes both PRP and control areas; paired comparison)	52 paired areas	51.2 (18.6)	31 (60)	Deep dermal/full-thickness burns	Gravitational Platelet Separation System III	Topical before STSG	STSG alone	1:1–1:9 Mesh / Meek	5–7 days (primary outcome); scar assessmen t up to 12 months
**Waiker 2015**	RCT	Parallel patient groups (patient-level	100/100	Not reporte d (age groups only)	140 (70)	Acute and chronic wounds including trauma, infection, burns, post-	Autologous PRP prepared by centrifugation (1000 rpm × 5 min) using	Topical before STSG	STSG + sutures/staple s	Not reporte d	3 months

		comparison)				burn contracture release	CPD-A anticoagulant				
**Sonker 2015**	Observational comparative study	Intra-patient (split-wound; same wound divided into PRP and control)	20 / 20	Not reported (range 5–60)	13 (65%)	Mixed wounds (burn contractures, trauma, ulcers, arteriovenous malformations)	Allogeneic PRP (apheresis, freeze–thaw activation)	Applied to half of wound bed	Conventional STSG (no PRP)	Not reported	42 days
**Gupta 2024**	RCT	Parallel-group (wounds allocated to PRP or control and analyzed as independent units)	40 / 40	40.4 (17.51)	44 (81.48%)	Mixed wounds (burns, trauma, fasciotomy wounds, vascular disease, ulcers)	Autologous PRP (centrifugation 3500 rpm, single spin method)	Applied over wound bed before graft placement	Conventional STSG (sutures alone)	Not reported	7 days

Table S2. Risk of bias assessment of randomized controlled trials (RoB 2)

Study / Year	Randomization process	Deviations from intended interventions	Missing outcome data	Outcome measurement	Selection of reported results	Overall risk of bias
**Dhua 2019**	Some concerns	Some concerns	Low	Some concerns	Some concerns	Some concerns
**Gupta 2020**	Some concerns	Some concerns	Low	Some concerns	Some concerns	Some concerns
**Hersant 2017**	Low	Some concerns	Low	Low	Some concerns	Some concerns
**Raguram 2022**	Some concerns	Some concerns	Low	Low	Some concerns	Some concerns
**Marck 2016**	Low	Low	Some concerns	Low	Low	Some concerns
**Waiker 2015**	Some concerns	Some concerns	Low	Some concerns	Some concerns	Some concerns
**Gupta 2024**	Some concerns	Some concerns	Low	Some concerns	Some concerns	Some concerns

Table S3. Risk of bias assessment of observational studies (ROBINS-I)

Study / Year	Bias due to confounding	Bias in selection of participants	Bias in classification of interventions	Bias due to deviations from intended interventions	Bias due to missing data	Bias in measurement of outcomes	Bias in selection of the reported result	Overall risk of bias
**Chigurupati 2024**	Serious	Moderate	Low	Moderate	Low	Moderate	Moderate	Serious
**Divyang 2025**	Moderate	Low	Low	Moderate	Low	Moderate	Moderate	Moderate
**Sonker 2015**	Serious	Moderate	Low	Moderate	Low	Moderate	Moderate	Serious

Table S4. GRADE assessment of certainty of evidence for outcomes

Outcome	Studies (RCTs)	Studies (Observational)	Risk of bias	Inconsistency	Indirectness	Imprecision	Publication bias	Certainty
Skin graft take	Gupta 2024<br>Hersant 2017<br>Marck 2016	Divyang 2025	Serious	Serious	Not serious	Serious	Not assessed	Very low
Hematoma	Gupta 2024<br>Dhua 2019<br>Gupta 2020<br>Waiker 2015	Chigurupati 2024<br>Divyang 2025	Serious	Not serious	Not serious	Serious	Not assessed	Low
Skin graft loss	Dhua 2019<br>Gupta 2020<br>Raguram 2022<br>Waiker 2015	Sonker 2015	Serious	Not serious	Not serious	Serious	Not assessed	Low

PRISMA 2020 Checklist

Section and Topic	Item #	Checklist item	Location where item is reported
**TITLE**			
Title	1	Identify the report as a systematic review.	1
**ABSTRACT**			
Abstract	2	See the PRISMA 2020 for Abstracts checklist.	1-2
**INTRODUCTION**			
Rationale	3	Describe the rationale for the review in the context of existing knowledge.	2-3
Objectives	4	Provide an explicit statement of the objective(s) or question(s) the review addresses.	2-3
**METHODS**			
Eligibility criteria	5	Specify the inclusion and exclusion criteria for the review and how studies were grouped for the syntheses.	4
Information sources	6	Specify all databases, registers, websites, organisations, reference lists and other sources searched or consulted to identify studies. Specify the date when each source was last searched or consulted.	4
Search strategy	7	Present the full search strategies for all databases, registers and websites, including any filters and limits used.	supplement
Selection process	8	Specify the methods used to decide whether a study met the inclusion criteria of the review, including how many reviewers screened each record and each report retrieved, whether they worked independently, and if applicable, details of automation tools used in the process.	5
Data collection process	9	Specify the methods used to collect data from reports, including how many reviewers collected data from each report, whether they worked independently, any processes for obtaining or confirming data from study investigators, and if applicable, details of automation tools used in the process.	5
Data items	10a	List and define all outcomes for which data were sought. Specify whether all results that were compatible with each outcome domain in each study were sought (e.g. for all measures, time points, analyses), and if not, the methods used to decide which results to collect.	5
	10b	List and define all other variables for which data were sought (e.g. participant and intervention characteristics, funding sources). Describe any assumptions made about any missing or unclear information.	5, Prospero Protocol
Study risk of bias assessment	11	Specify the methods used to assess risk of bias in the included studies, including details of the tool(s) used, how many reviewers assessed each study and whether they worked independently, and if applicable, details of automation tools used in the process.	5
Effect measures	12	Specify for each outcome the effect measure(s) (e.g. risk ratio, mean difference) used in the synthesis or presentation of results.	6
Synthesis methods	13a	Describe the processes used to decide which studies were eligible for each synthesis (e.g. tabulating the study intervention characteristics and comparing against the planned groups for each synthesis (item #5)).	6
	13b	Describe any methods required to prepare the data for presentation or synthesis, such as handling of missing summary statistics, or data conversions.	6

Section and Topic	Item #	Checklist item	Location where item is reported
	13c	Describe any methods used to tabulate or visually display results of individual studies and syntheses.	6
	13d	Describe any methods used to synthesize results and provide a rationale for the choice(s). If meta-analysis was performed, describe the model(s), method(s) to identify the presence and extent of statistical heterogeneity, and software package(s) used.	6
	13e	Describe any methods used to explore possible causes of heterogeneity among study results (e.g. subgroup analysis, meta-regression).	6
	13f	Describe any sensitivity analyses conducted to assess robustness of the synthesized results.	6
Reporting bias assessment	14	Describe any methods used to assess risk of bias due to missing results in a synthesis (arising from reporting biases).	6
Certainty assessment	15	Describe any methods used to assess certainty (or confidence) in the body of evidence for an outcome.	6
**RESULTS**			
Study selection	16a	Describe the results of the search and selection process, from the number of records identified in the search to the number of studies included in the review, ideally using a flow diagram.	7
	16b	Cite studies that might appear to meet the inclusion criteria, but which were excluded, and explain why they were excluded.	7
Study characteristics	17	Cite each included study and present its characteristics.	7, supplement
Risk of bias in studies	18	Present assessments of risk of bias for each included study.	12, supplement
Results of individual studies	19	For all outcomes, present, for each study: (a) summary statistics for each group (where appropriate) and (b) an effect estimate and its precision (e.g. confidence/credible interval), ideally using structured tables or plots.	9-13, supplement
Results of syntheses	20a	For each synthesis, briefly summarise the characteristics and risk of bias among contributing studies.	9-13, supplement
	20b	Present results of all statistical syntheses conducted. If meta-analysis was done, present for each the summary estimate and its precision (e.g. confidence/credible interval) and measures of statistical heterogeneity. If comparing groups, describe the direction of the effect.	9-13, supplement
	20c	Present results of all investigations of possible causes of heterogeneity among study results.	9-13, supplement
	20d	Present results of all sensitivity analyses conducted to assess the robustness of the synthesized results.	9-13, supplement
Reporting biases	21	Present assessments of risk of bias due to missing results (arising from reporting biases) for each synthesis assessed.	9-13, supplement
Certainty of	22	Present assessments of certainty (or confidence) in the body of evidence for each outcome assessed.	12-13,

Section and Topic	Item #	Checklist item	Location where item is reported
evidence			supplement
**DISCUSSION**			
Discussion	23a	Provide a general interpretation of the results in the context of other evidence.	13-15
	23b	Discuss any limitations of the evidence included in the review.	13-15
	23c	Discuss any limitations of the review processes used.	13-15
	23d	Discuss implications of the results for practice, policy, and future research.	13-15
**OTHER INFORMATION**			
Registration and protocol	24a	Provide registration information for the review, including register name and registration number, or state that the review was not registered.	4
	24b	Indicate where the review protocol can be accessed, or state that a protocol was not prepared.	4, Reference [10]
	24c	Describe and explain any amendments to information provided at registration or in the protocol.	4
Support	25	Describe sources of financial or non-financial support for the review, and the role of the funders or sponsors in the review.	15
Competing interests	26	Declare any competing interests of review authors.	15
Availability of data, code and other materials	27	Report which of the following are publicly available and where they can be found: template data collection forms; data extracted from included studies; data used for all analyses; analytic code; any other materials used in the review.	15

From: Page MJ, McKenzie JE, Bossuyt PM, Boutron I, Hoffmann TC, Mulrow CD, et al. The PRISMA 2020 statement: an updated guideline for reporting systematic reviews. BMJ 2021;372:n71. doi: 10.1136/bmj.n71. This work is licensed under CC BY 4.0. To view a copy of this license, visit https://creativecommons.org/licenses/by/4.0/
